# Some Scientific Aspects of Insanity

**Published:** 1892-03-26

**Authors:** 


### Some Scientific Aspects of Insanity.

### XVIII.—CLIMACTERIC INSANITY.

As the influx of new life and new ideas at adolescence is frequently attended in those whose nervous systems are unstably constituted by serious mental aberrations, so at the climacteric the ebb and decadence of those same reproductive functions is also in persons of unstable brain liable to be attended by mental disturbances. The climacteric period is not an exact one, but varies with the individual, and extends indefinitely from one to three years. Even in the sanest people it is attended by a change in the mental disposition in the affectiveness which we observed to be one of the characteristics of the adolescent period. It is with an individual at the time of the climacteric as it is with a house in which the fires have gone out. The house remains a good house, but the sparkle and the light is wanting. The subtle all-pervading instinct of sex which is such a potent factor in the individual, and in society is visibly and sensibly weakened and lessened, and with its decay the individual loses a large portion of that exhiliration which for thirty years has leavened his life. Along with those mental changes there are frequently physical changes. The bodily configuration, especially in the female sex, alters considerably to the extent of visibly diminishing those outward signs, which are usually associated with vigorous sexual life in woman. But besides this, the vital and trophic functions of the body are slackened, and become slower.

From what has been said, it might be expected that the form of mental disease which supervenes at this period is melancholia, which disease is, as we have already seen, the expression of lowered nervous activity. Melancholia, then, both in men and women, is almost with no exception the characteristic mental affection of the climacteric.

Every degree of melancholia is met with at this period, varying from slight attacks not requiring asylum treatment, up to those which are so severe as often to be intractable under the ordinary methods of treatment. The most typical melancholias are met with among patients at this period. Chiefly because the more haggard, careworn expression lends itself better to the representation of the morbid mental condition. There is also a greater tendency to dark pigmentation of the skin, to pallor of the face, and to dryness of the hair; while the want of elasticity in the carriage permits in a greater degree of the feeble, stooping gait which one naturally associates with the idea of melancholia. Many patients who have been affected at the adolescent period are liable again to be attacked with mental disturbance at the climacteric, and it is in them especially that the melancholia is often so prolonged, so incurable, and so distressing. Those neurotic subjects who have been overtaken with mental disease at the adolescent period, and again, if they are women at the puerperal period, are apt to find in climacteric insanity the grave of their mental faculties. There are two chief reasons why the melancholia at this time of life is so difficult to overcome. One is that the buoyant feeling of well-being, generally present in youth, and which occasionally shines out during the course of the very worst cases of melancholia at a younger age is here absent. The other reason is that the element of hope for the future, so strong in the youthful mind, is towards the end of life apt to be diminished, and there is, therefore, no outlook for the patient but his own gloomy forebodings. Where nature does not aid medicine cure is less possible, and it is because the natural flame of manhood and womanhood burns less brightly at the climacteric, that climacteric insanity is often so difficult to relieve. The methods at our disposal for the treatment of melancholia are of course the only means that can be used in the alleviation of this special form, and these have been already described.

Those cases with a strong neurosis and an hereditary tendency to insanity, and which, moreover, have suffered from that disease at one or other of the three periods to which we have referred, are not unlikely to succumb to an early senility.

We thus see that in insanity, as in every other disease, there are two causative factors to be considered, an inherent tendency to weakness of the nervous system, and the strains and stresses of life, chief among which are the crises of the reproductive function.

The 34th annual meeting of the Dental Hospital of London was held on the 10th inst. In the report, which was unanimously adopted, the Managing Committee congratulated the Governors on the continued success of the institution; also on the great benefits which the hospital continues to afford to the suffering poor, 54,177 cases having been treated during the year 1891, a large number of them painlessly (under anæsthetics), being 5,413 over the previous year, and 34,922 in excess of the number treated in 1874, when the hospital was removed to its present site. In consequence of the still increasing number of patients, it had been decided that in addition to the morning all departments of the hospital should be open in the afternoon. The dental appliance department, in connection with supplying artificial teeth, &c., was still doing good work on behalf of the charity. That, unfortunately, there was £1,000 remaining of the mortgage debt on the hospital, and they urgently appealed for funds to enable them to rid themselves of this encumbrance, incurred for the extension of the hospital, rendered indispensably necessary to meet the growing wants of the charity. It was most desirable, if the good work of the institution is to continue, that new annual subscribers should be introduced. The charity is unendowed, and additional funds would enable it to greatly extend its usefulness.

